# Primary cutaneous cryptococcosis – History, concepts, clinical and therapeutic update^☆^

**DOI:** 10.1016/j.abd.2024.07.004

**Published:** 2024-11-15

**Authors:** Sílvio Alencar Marques, Rosangela Maria Pires de Camargo

**Affiliations:** Department of Infectology, Dermatology, Diagnostic Imaging and Radiotherapy, Faculty of Medicine, Universidade Estadual Paulista, Botucatu, SP, Brazil

**Keywords:** Clinical medicine, Cryptococcosis, Cryptococcosis/diagnosis, Cryptococcosis/therapy, Mycology

## Abstract

Cryptococcosis is a disease caused by fungi of the genus *Cryptococcus*, with the species *Cryptococcus neoformans* and *Cryptococcus gattii* being recognized as pathogenic. Cutaneous cryptococcosis can be classified as “secondary”, developing from a previous systemic disease, or, on the contrary, “primary”, resulting from transcutaneous inoculation of the agent. It can also be classified as “disseminated cutaneous cryptococcosis”, when there is an associated systemic disease, or “localized”, when it is restricted to the skin. This article uses the term “primary cutaneous cryptococcosis” because it is the most widely used and already established in the literature. Historically, the first report of a possible case of primary cutaneous cryptococcosis (PCC) occurred in 1950 by Gancy WM and was published in the Archives of Dermatology and Syphilology. Subsequently, the rare and sporadic reports in the following decades were reviewed and reported in the 1985 publication by Baes & van Cutsen. However, the unequivocal acceptance of the existence of PCC as a distinct disease only occurred in 2003 with the publication by Neville S et al. of the French Cryptococcosis Study Group. The fundamental criterion established to consider it as PCC was the proven absence of systemic disease, whether pulmonary, in the CNS or other location at the time of diagnosis of the cutaneous condition, characterized by a single lesion and, mostly, in an exposed area. These and other clinical criteria, diagnostic confirmation, and therapeutic choice are discussed in detail in the full text.

## Cryptococcosis. Etiopathogenesis. Concepts. Classification

Cryptococcosis is a human and animal disease caused by fungi of the genus *Cryptococcus*, an encapsulated yeast with several species, of which the recognized pathogens are *Cryptococcus neoformans* and *Cryptococcus gattii*. *C. neoformans* is a cosmopolitan, universal fungus, frequently isolated from pigeon droppings, decaying vegetables and fruits, and causes human disease predominantly in immunocompromised patients.^1^, ^2^ On the other hand, *C. gattii* has a more regionalized distribution, prevailing in tropical and subtropical regions, although it has been isolated in temperate regions. It should be noted that *C. gattii* infects both immunocompromised and immunocompetent patients.^3^, ^4^ *C. gattii* is isolated from soil samples, decaying vegetables, and tree hollows, and has a special ecological association with *Eucalyptus camaldulensis* plantations.^3^, ^5^ In 2015, Hagen F et al.^6^ published an article on sufficient molecular evidence to subdivide *C. neoformans* into two new species and *C. gattii* into four additional new species, namely: *C. neoformans* and *C. deneoformans*; and, the species *C. gattii*, *C. deuterogattii*, *C. bacillosporus*, *C. tetragattii* and *C. decagattii* and also the possibility of hybrid species.^6^ This proposal, although based on multiple molecular data, did not find support among experts, including Kwon-Chung KJ et al.^7^ The latter authors, under various arguments, including the lack of association of specific clinical manifestations with a particular subspecies, propose the use of the name *C. neoformans* complex (*Cryptococcus neoformans species complex*) and *C. gattii* complex (*Cryptococcus gattii species complex*).^7^ This suggestion has been followed by several authors in recent reports.^8^, ^9^, ^10^

Cutaneous cryptococcosis can be classified as “secondary” to dissemination from a previous systemic disease or “primary”, by transcutaneous inoculation of the agent. Or, it can be classified as “disseminated cutaneous cryptococcosis”, that is, resulting from systemic disease with a cutaneous lesion originating from hematogenous dissemination of the fungus. Or, on the contrary, it can be classified as “localized”, when restricted to the skin.^11^ In this article, the term “primary cutaneous cryptococcosis” is used, following the concept of local transcutaneous inoculation, as it is more widely used and already established in the literature.^12^

Classically, in systemic disease, the natural history of cryptococcosis results from inhalation of the fungi and a pulmonary condition that is almost always asymptomatic or oligosymptomatic. In terms of evolution, immediate hematogenous dissemination or the permanence of a quiescent pulmonary focus and subsequent hematogenous dissemination due to an imbalance in the agent-host relationship may occur.^11^ It is a typical disease of immunocompromised individuals and is considered, in itself, a defining factor for AIDS in HIV-infected individuals, as well as an incidental disease in patients after solid organ transplantation or on prolonged corticosteroid therapy.^13^ Cryptococcosis in immunocompromised patients presents most frequently as involvement of the central nervous system (CNS), with initially indolent manifestations and progressive evolution to severe and potentially fatal meningoencephalitis. An isolated or predominant pulmonary clinical manifestation is not common, but possible. Other organs and systems may be affected, including the skin in up to 15% of AIDS cases; therefore, producing a secondary skin lesion which functions as sentinel of associated systemic involvement.^11^, ^13^ The dermatological clinical presentation of a lesion associated with a systemic focus is distinct from that resulting from a primary skin lesion and therefore helps in the diagnostic differentiation between “primary” or “secondary” cutaneous cryptococcosis. When the skin lesion is secondary to systemic disease, the most frequent clinical manifestation are multiple lesions, mainly in the cephalic segment, with possible mucosal lesions, albeit uncommon.^11^, ^14^, ^15^ In contrast, the primary cutaneous lesion is a single, polymorphic lesion, most often an infiltrated plaque of varying extent, with a tendency to necrosis and no mucosal involvement.^11^, ^12^

## Primary cutaneous cryptococcosis

### History

Sporotrichosis and chromoblastomycosis are mycoses classically accepted as examples of subcutaneous implantation mycoses and primary transcutaneous infection. Other mycoses, which are classically systemic and inhalation-mediated infections, such as coccidioidomycosis, blastomycosis, histoplasmosis, and even paracoccidioidomycosis, may exceptionally be the result of transcutaneous inoculation.^16^, ^17^ However, these events, in these circumstances, constitute rare, exceptional cases and are a clear exception to the rule. The first report of a possible case of primary cutaneous cryptococcosis (PCC) occurred in 1950 by Gancy WM and was published in the Archives of Dermatology and Sypholology.^18^ This report was followed by rare and sporadic publications in the following decades, with the reported cases associated or not with immunocompromised patients.^18^, ^19^, ^20^, ^21^ These publications were reviewed and reported in detail by Baes & van Cutsen in 1985.^22^ However, the unequivocal acceptance of the existence of PCC as a clinical subtype distinct from classical cryptococcosis and recognized as such occurred in 2003 with the publication of Neville S et al.^23^ of the French Cryptococcosis Study Group. In this publication, the authors compared data from 28 patients with a presumptive diagnosis of PCC with those from 80 cases of disseminated classical cryptococcosis with skin lesions and also data from 1866 cases of disseminated cryptococcosis and involvement of organs other than the skin.^23^ The basic criterion for considering a presumptive diagnosis of PCC was, evidently, the proven absence of systemic disease, whether pulmonary, in the central nervous system or any other location at the time of diagnosis of the skin condition. Among the various differences observed in the groups studied above, the semiological pattern and location of the lesions were differentiated. The cases of PCC were restricted to single lesions located mainly in exposed areas, sparing the cephalic segment. On the other hand, the cutaneous lesions secondary to systemic disease were almost always multiple, papulonodular, often molluscum contagiosum-like and preferentially located in the cephalic segment. It is worth noting that the same article shows the occurrence of PCC cases in immunocompetent patients, which did not occur in the systemic disease cases studied therein. Therefore, based on this explicit demonstration that these are distinct clinical expressions, the concept of “primary cutaneous cryptococcosis” was consolidated.^23^ The criteria that favor suspicion and determine the definitive diagnosis of one or the other form of the disease are didactically and clearly demonstrated in tables in this reference article by Neville S et al.^23^

### Diagnostic criteria. Clinical history and clinical-dermatological examination

The clinical history of the current disease in PCC is variable, ranging from weeks to months. There is not always a history of trauma preceding the lesion, just as there is not always a history of exposure to bird or bat droppings or rural life. The lesions are almost always located in exposed areas subject to trauma, particularly the arm and forearm. The complaint may be of pain and/or local heat. There is no complaint of fever, systemic signs or symptoms. There is often a history of immunosuppressive medication use for different reasons: post-solid organ transplant, corticosteroid therapy for chronic obstructive pulmonary disease, autoimmune diseases, particularly rheumatic joint disease, use of biological immunosuppressants or chemotherapy. When affecting the elderly, even in the absence of medication or an evident cause of immunocompromise, immunosenescence, in itself, may be the contributing factor to be considered.

**Figure 1:**
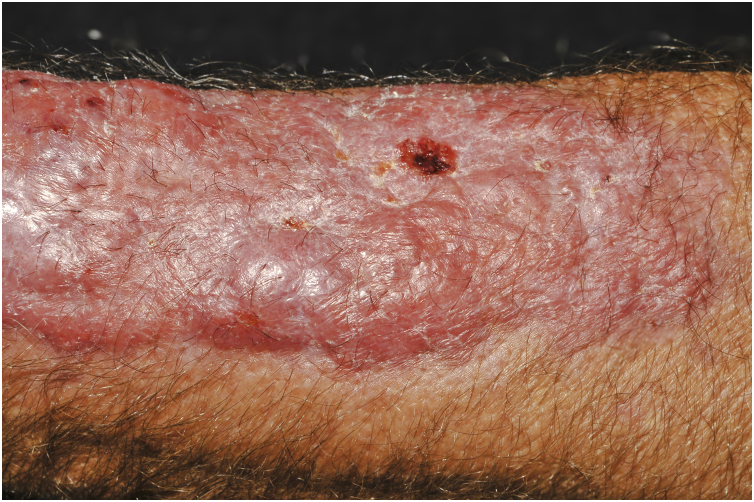
Primary cutaneous cryptococcosis (PCC): Infiltrated, erythematous plaque with raised surface, showing a tumor-like aspect. Rare ulcerated or necrotic spots. Forearm of an immunocompetent 66-year-old male patient, post-trauma.

**Figure 2:**
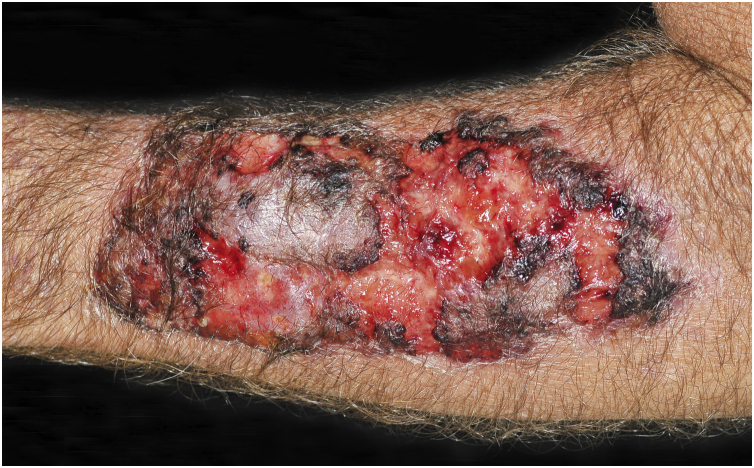
PCC: Ulceronecrotic lesion with precise limits, absence of inflammatory halo. Forearm of an immunocompetent 58-year-old male patient, post-trauma.

**Figure 3:**
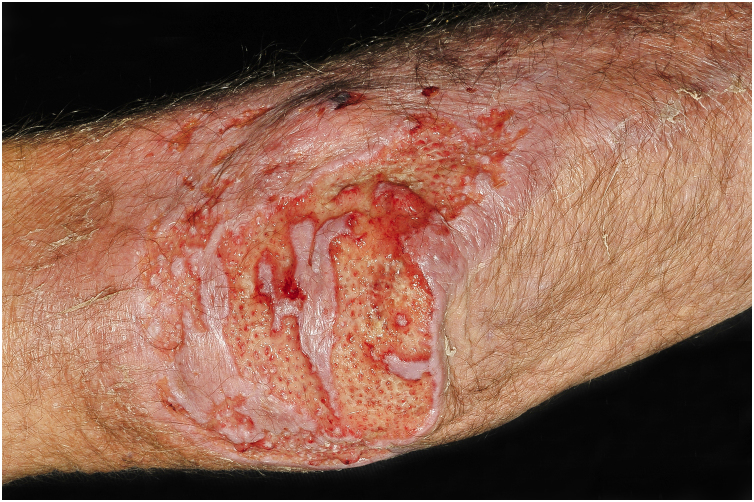
PCC: Ulcerated lesion, clear background, with hemorrhagic spots, islands of intact tissue and infiltrated borders. The photo was taken after debridement. Elbow of a 74-year-old male patient with COPD, on irregular corticosteroid therapy, 10 mg/day. Post-trauma lesion, which occurred during the cleaning of the attic of a Catholic church.

**Figure 4:**
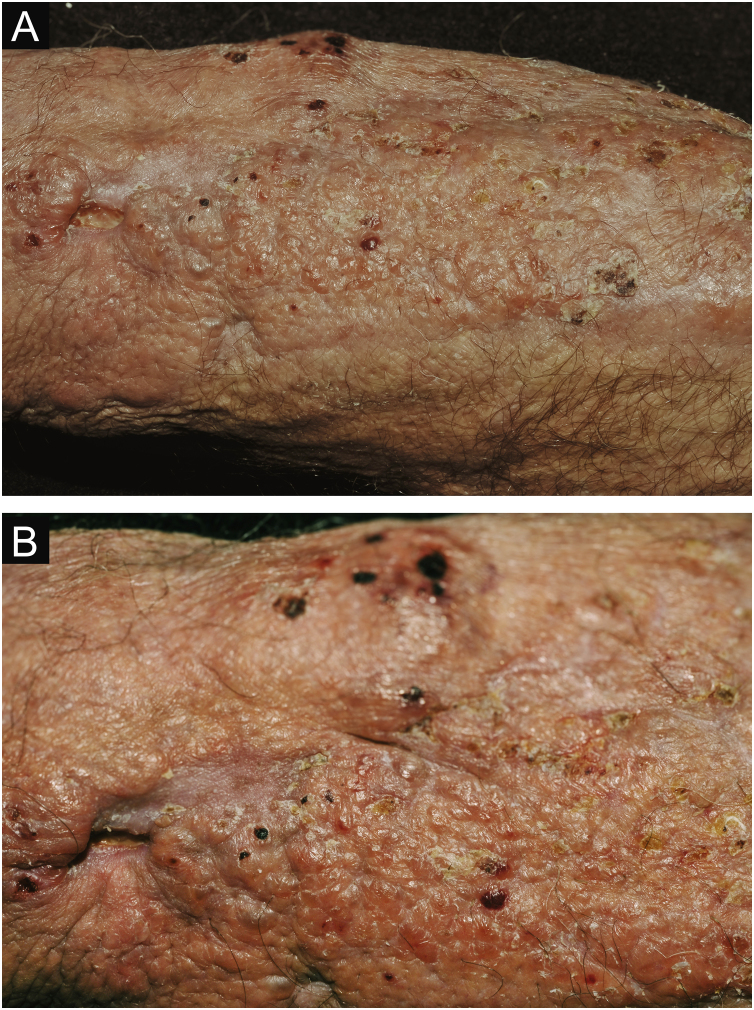
PCC: (A) Extensive plaque with necrotic, ulcerated spots and pseudovesicles crusts in a 70-year-old male patient, on irregular corticosteroid therapy 5 mg/day, due to arthritis. The patient lived in a rural area. (B) Detail: Presence of a tumor-like lesion with necrotic spots and satellite lesions with pseudovesicles.

**Figure 5:**
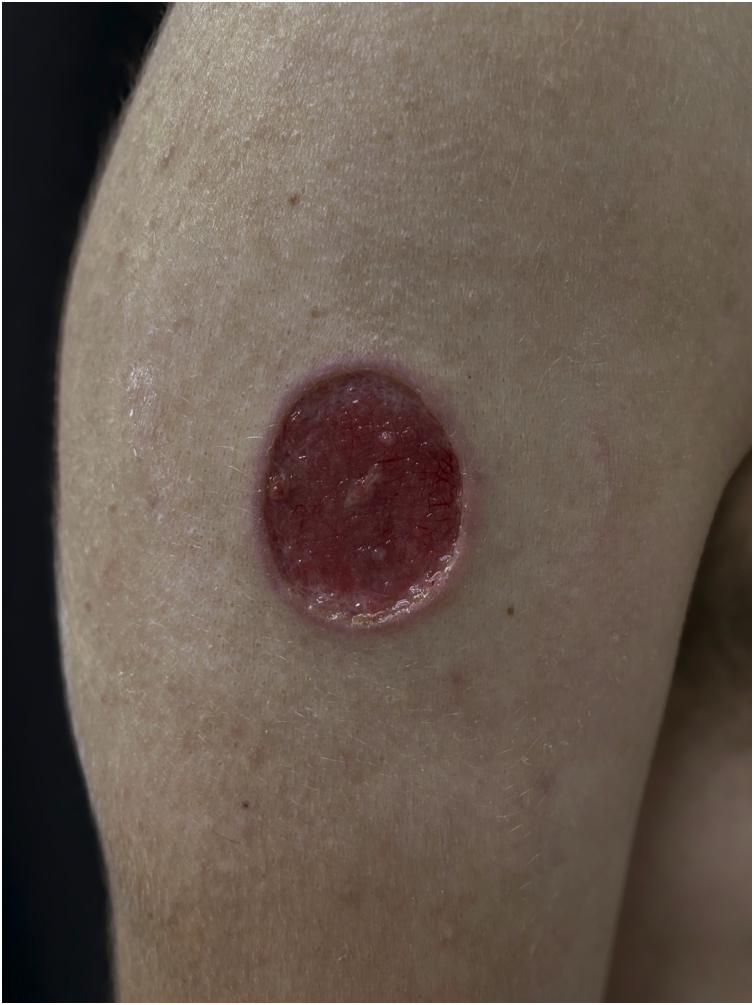
PCC: Ulcerated lesion, granular background, with borders framing the lesion in a leishmaniasis-like appearance on the deltoid region of a 31-year-old male patient, a kidney transplant recipient after seven years. The patient was receiving prednisone 5 mg and tacrolimus 0.2 mg/kg/day.

**Figure 6:**
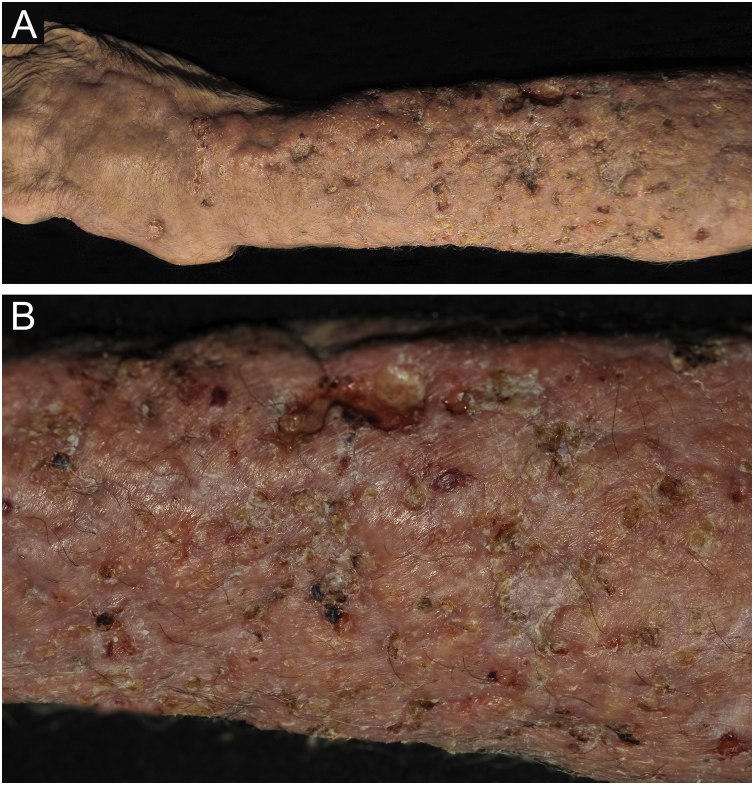
PCC: (A) Infiltrated, extensive lesion, showing nodules, ulcerations, necrotic spots, in the distal region of the arm, forearm and dorsum of the hand of a 77-year-old male patient with COPD, on corticosteroid therapy, 10 mg/day. The patient lived in a rural area. (B) Detail: Nodules, scar retractions, ulceration spots and necrosis.

**Figure 7:**
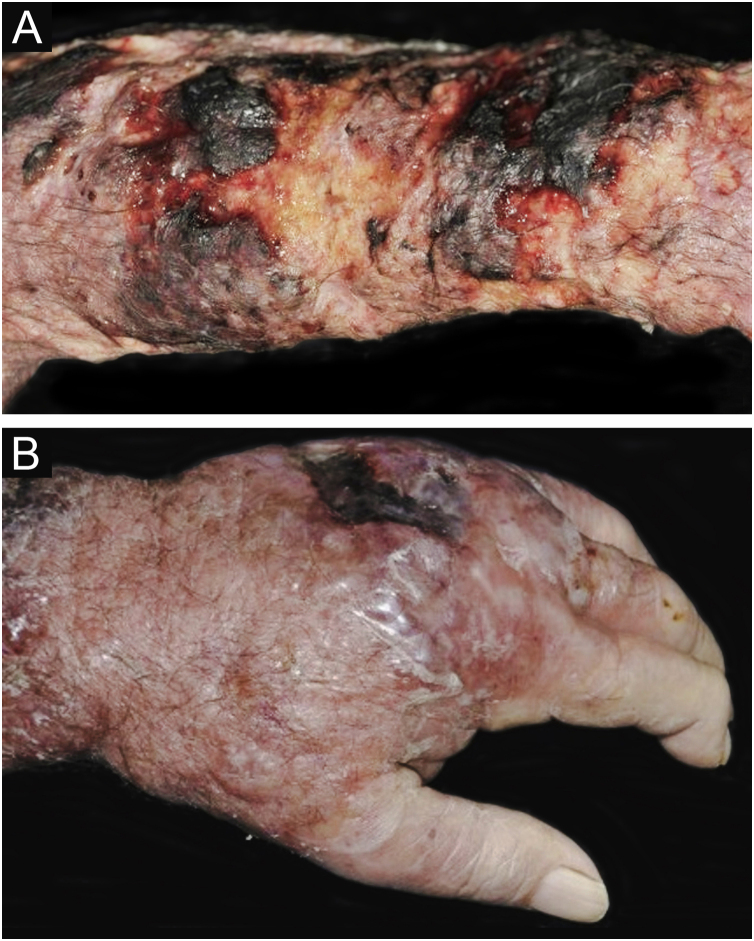
CCP: (A) Extensive phagedenic and terebrant lesion, with necrotic and ulcerated areas, on the arm, forearm and dorsum of the hand of an 80-year-old male patient on irregular use of corticosteroid therapy 10 mg for years, due to arthritis. (B) Erythematous-edematous lesion, with vesicles and bullae, and ​​necrotic area on the dorsum of the hand (Patient from Reference #12).

Clinical suspicion of PCC arises from a single skin lesion located in an exposed area, particularly the upper limbs, with acute or subacute progression, with compatible dermatological clinical characteristics and no signs or symptoms of systemic disease. Semiologically, the lesion is polymorphic, most often infiltrative, tumor-like, ulcerated or not, occasionally leishmaniasis-like, with or without necrotic spots, and rarely cellulitis-like. The lesion diameter is variable and may affect an entire segment of the affected limb, and lesion limits are not always precise. The coloration is erythematous, erythematous-violet, or erythematous-brownish (Figure 1, Figure 2, Figure 3, Figure 4, Figure 5).^11^, ^12^, ^23^, ^24^, ^25^, ^26^, ^27^ On palpation, the consistency may be firm or somewhat soft, gelatinous, due to the large number of fungal elements, their mucoid capsules, and the scarce inflammatory response.^12^ Bacterial coinfection and local inflammatory signs are not uncommon. If the diagnosis is delayed, the lesion tends to develop a large diameter, with skin surface necrosis (Figure 6, Figure 7). There are multiple differential diagnoses and the use of the PLECT syndromic diagnosis (paracoccidioidomycosis, leishmaniasis, sporotrichosis [*Esporotricose* – portuguese language], chromoblastomycosis and cutaneous tuberculosis) is recommended as an initial guide. The hypotheses of pyoderma gangrenosum, severe bacterial infections, ecthyma gangrenosum including their possible causal agents, and herpes zoster with necrosis should be added to the clinical reasoning in immunocompromised patients. Given the acute or subacute nature of the process, neoplastic diseases are not plausible differential diagnoses.

It is not unusual for the initial suspicion to be of a bacterial or even viral infectious process (herpes zoster) and only after therapeutic failure with the use of antibiotics or antivirals or even worsening while taking them, does the attending physician submit the lesion to histopathological or direct mycological examination. Once the diagnosis of cutaneous cryptococcosis has been established, confirmation of whether it is primarily cutaneous and of the infecting species must follow the academically well-established criteria and steps briefed below.

### Diagnostic confirmation

**Figure 8:**
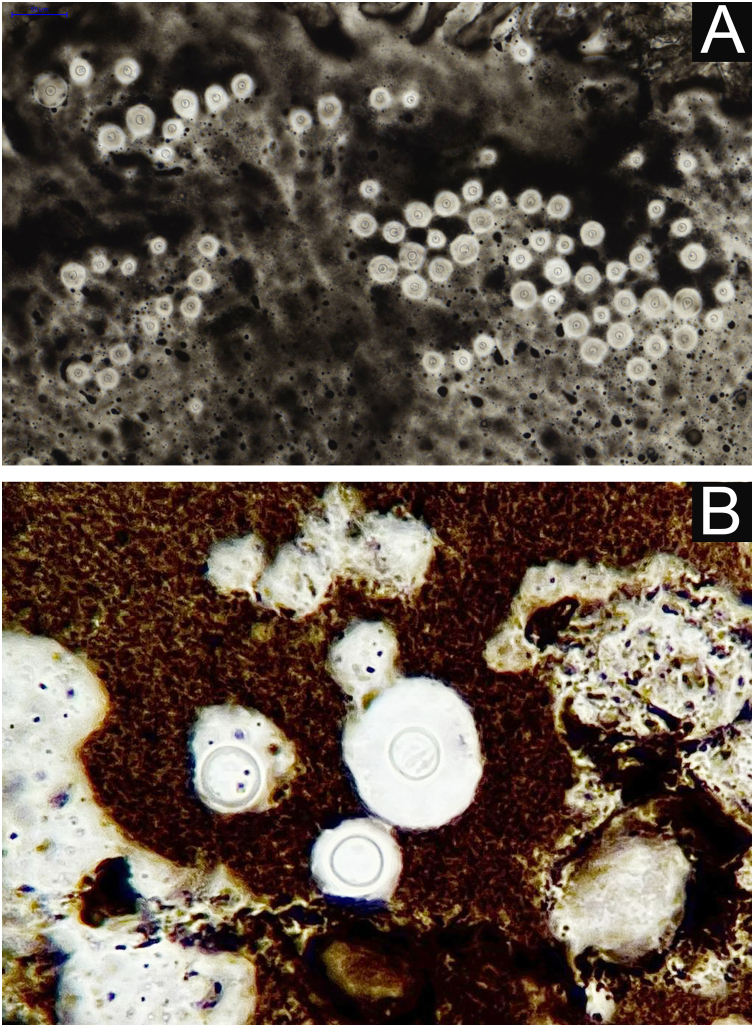
PCC: Direct mycological examination. (A) Indian ink. (B) Detail: fungal cell with mucoid capsule highlighted by contrast with the black color of Indian ink.

Diagnostic confirmation is based on the analysis of several criteria:

**I** - Observation of the agent on direct examination (direct mycological examination). A sample obtained from the lesion by biopsy, curettage, or scraping with a blunt scalpel is gently smeared on a blood count slide, to which drops of Indian ink (or Nanking ink) are added. Or, even in the absence of Indian ink, a smear of a lesion sample treated with drops of saline solution can be used. India ink or saline solution do not penetrate the mucoid capsule of *Cryptococcus sp*. and, as a result, the capsule stands out, contrasting with the black of the India ink or the pink of the hemorrhagic medium (Fig. 8).^28^

**Figure 9:**
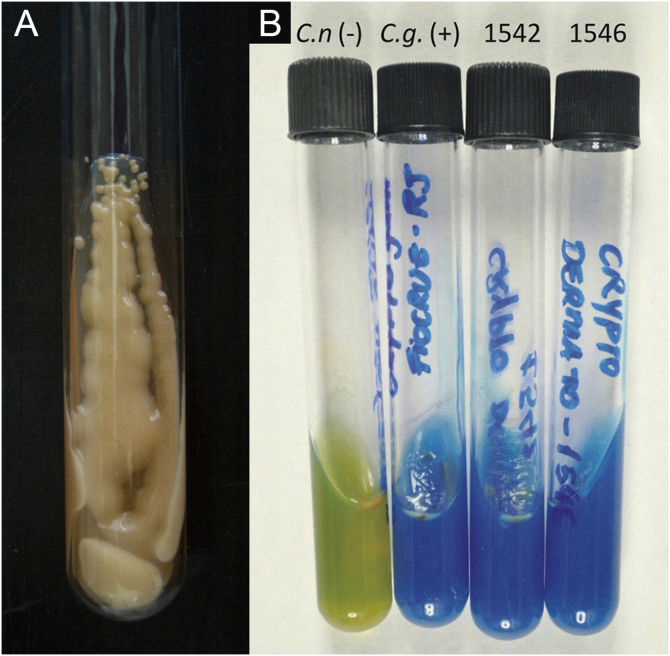
PCC: (A) *Cryptococcus* spp. Yeast culture; appearance of condensed milk. (B) *Cryptococcus neoformans* and *C. gattii*. CGB Agar. Yellowish-green tube, original CGB agar– *C. neoformans*. Cobalt blue tube, modified CGB agar – *C. gattii*.

**II** – Culture with seeding on Niger seed agar or Sabouraud dextrose agar. Mycosel agar should not be used, as it contains cycloheximide (Actidione®), which inhibits the growth of several species of *Cryptococcus*.^28^, ^29^ The lesion sample for seeding can be obtained by micro biopsy or curettage of the lesion bed. The sample must be collected with maximum antisepsis, avoiding bleeding if possible, and sent to the mycology laboratory as soon as possible in a sterile bottle containing saline solution. The seeded test tube or Petri dish should ideally be kept at 30°‒32 °C, but under routine conditions they can be kept at room temperature. The growth of *Cryptococcus spp*. is rapid, already visible macroscopically after two to five days, with a yeast-like, creamy or creamy-white color, resembling condensed milk. If seeded on Niger seed medium, it shows a brownish-coffee pigmentation.^28^, ^29^ When seeded in a test tube, immobilized in a vertical position, the culture tends to “run” to the bottom of the tube (Fig. 9A).^28^

**III** ‒ Species definition can be obtained from a sample of the original, primary culture, in Niger seed or Sabouraud dextrose medium, and then transplanted onto a medium containing canavanine, glycine, and bromothymol blue (CGB agar). In this method, the original CGB agar has a yellow-green color, but when the transplanted culture is of the *Cryptococcus gattii* complex, the color of the agar changes to cobalt blue in two/three to five days. This color change occurs due to the degradation of creatine into ammonia and a change in the pH of the original CGB agar (pH 5) to an alkaline one (pH 7). Species of the *Cryptococcus neoformans* complex do not produce such changes and the CGB agar remains with its original yellow-green color (Fig. 9B). It is important to know that CGB agar is not indicated for obtaining the primary culture and is only used to differentiate between species.^30^, ^31^ A positive culture is considered the reference evidence for the diagnosis and is essential for academic and research purposes.

**IV** – Confirmation can be achieved through increasingly popular and widely used technologies such as automated auxanograms, which use samples from the primary culture, and also the use of the proteomic identification technique (MALDI Tof MS). These methods are available in commercial reference laboratories and better-equipped university hospitals. These are methods that provide fast, reliable results and identify species belonging to the *C. neoformans* or *C. gattii* complexes.

**V** – Molecular method: Through extraction and amplification of fungal DNA by polymerase chain reaction (PCR) and sequencing of the Internal Transcribed Spacer (ITS) of ribosomal DNA. Species identification, whether *Cryptococcus neoformans* complex or *Cryptococcus gattii* complex, results from the use of specific primers in the amplification and identification of serotypes. The molecular method, although very important from an academic point of view, is restricted to research and available in a few reference laboratories.^32^

**Figure 10:**
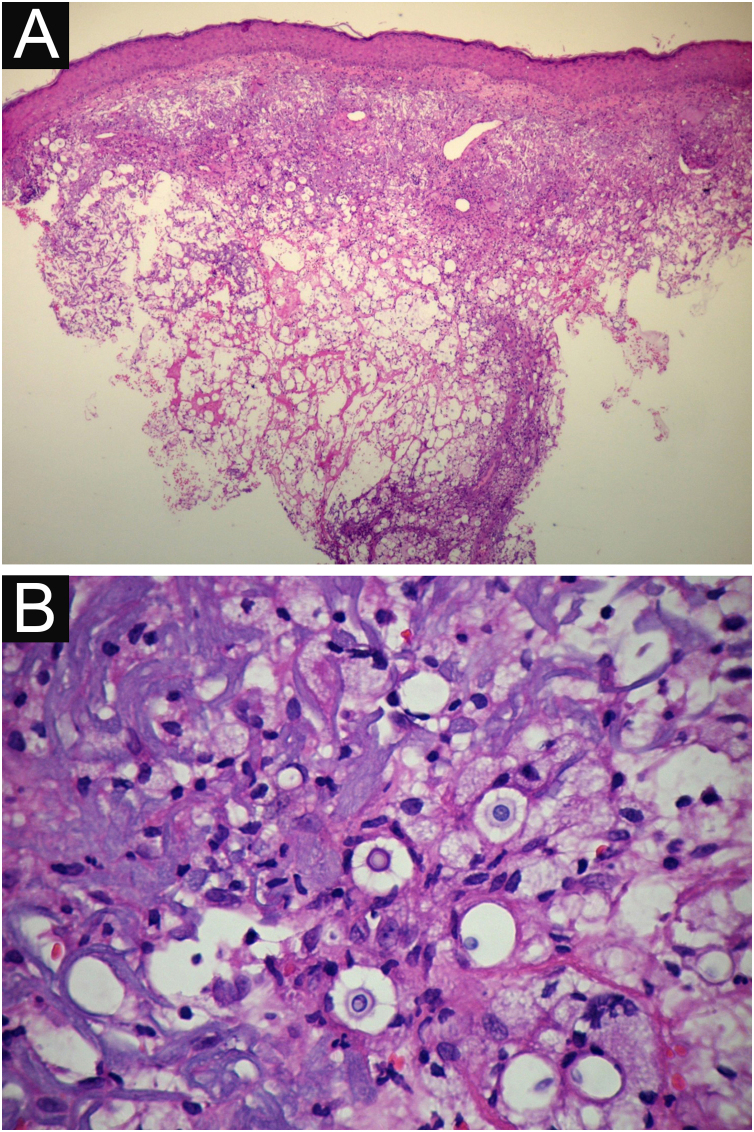
PCC: (A) Histopathology. Slightly acanthotic epidermis and dermal inflammatory infiltrate, surrounding a large clear area. (Hematoxylin & eosin ×40). (B) Detail showing histiocytic and lymphocytic infiltrate, basophilic degeneration of collagen and fungal cells with basophilic central nucleus and mucoid capsule (Hematoxylin & eosin ×200).

**Figure 11:**
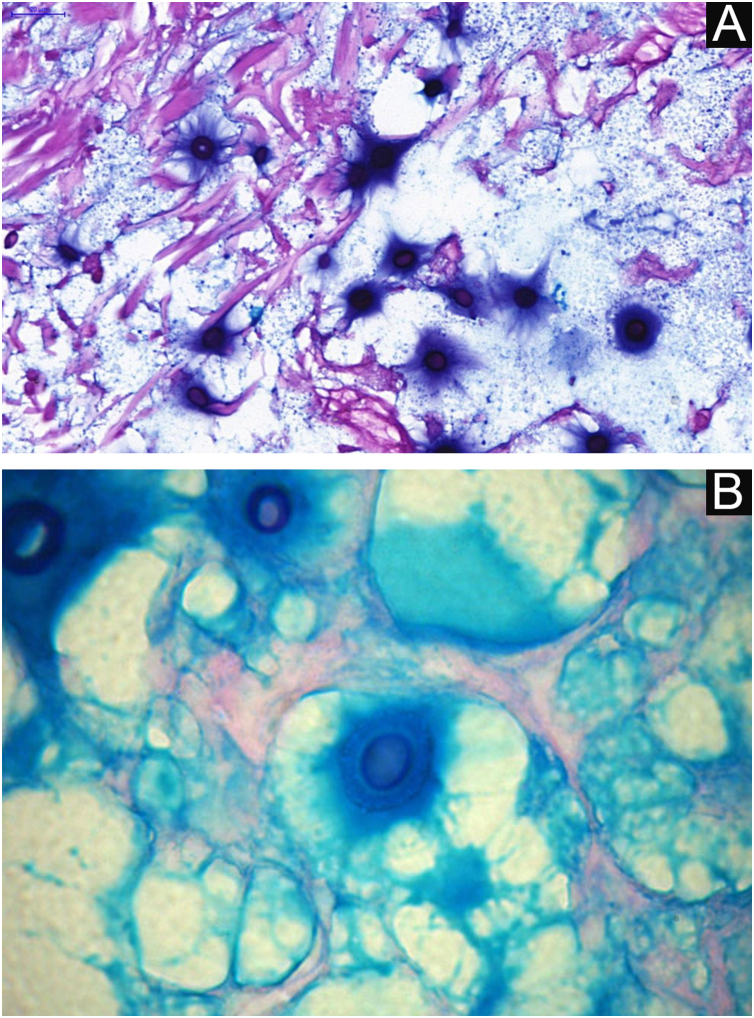
PCC – (A) Alcian blue staining showing *Cryptococcus* stained in blue, including the mucoid capsule. (B) High magnification detail.

**Figure 12:**
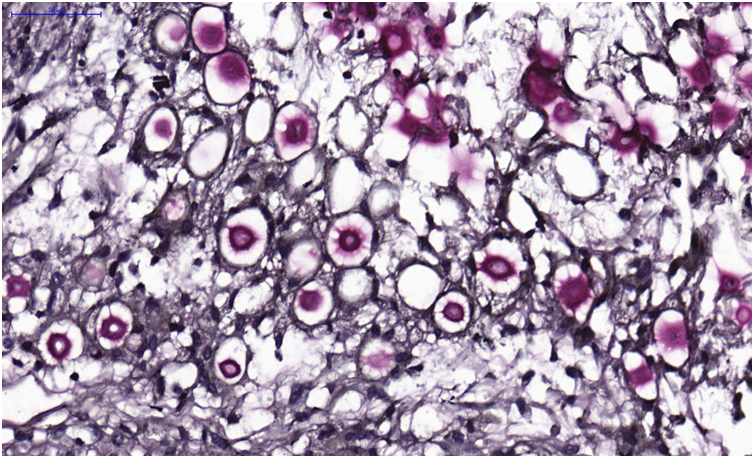
PCC: Mucicarmine staining showing *Cryptococcus* stained in dark red color including part of the mucoid capsule.

**VI** – Histopathological examination of a 5-punch or spindle-shaped biopsy with a scalpel is indicated, avoiding necrotic areas. The sample should be sent to the pathology laboratory immersed in 10% buffered formalin. Histopathological findings are characterized by moderate epidermal hyperplasia, with possible ulceration or necrosis foci, and scarce chronic inflammatory infiltrate in the dermis. Histiocytes and rare or few giant cells are observed. The infiltrate contains a variable number of lymphocytes, eosinophils, and neutrophils. The hematoxylin and eosin staining shows clear spaces in the dermis that correspond to areas occupied by numerous fungal elements producing “mucoid masses” irregularly permeated by the inflammatory infiltrate. The fungi are visible with the hematoxylin and eosin staining and are highlighted and more characteristically recognized with the mucicarmine and alcian-blue stainings, as these are capable of staining the mucoid capsule of the fungus. *Cryptococcus* species are also stained with periodic acid-Schiff (PAS) and silver-methenamine (Grocott-Gomori method). The Grocott-Gomori staining, in particular, allows the observation and quantification of the large number of fungal elements, characterized by single cells, stained in black, measuring 5 to 15 µm in diameter, contrasting with the greenish or yellowish background. Fungal cells usually present no buds or only one, but two buds are possible and, rarely, the presence of hyphae.^33^ PAS and Grocott-Gomori stains reveal the presence of fungal elements but do not allow absolute histological certainty in the diagnosis of cryptococcosis (Figs. 10‒12).^34^, ^35^ Non-encapsulated variants or those with small capsules, which are more difficult to recognize, should be considered, and it is suggested that they are associated with greater pathogenicity and greater inflammatory response.^36^, ^37^

**VII** – The serological method: by latex agglutination to detect the polysaccharide capsular antigen of *Cryptococcus sp*. It is performed in blood, cerebrospinal fluid, bronchoalveolar lavage, or urine samples. This method, in such samples, has a sensitivity of 95% and a specificity of 98%, in which titers >1/4 suggest infection and >1/8 suggest active disease. When PCC is suspected, the objective is to determine that, at least in serum and cerebrospinal fluid samples, the latex agglutination test is negative, which would indicate the absence of systemic involvement with 98% certainty.^38^ Also, the cryptococcal antigen-lateral flow immunochromatographic assay (CrAg-LFA), has been used for detection of the capsular antigen.^39^

**VIII** – Complementary tests: investigation of systemic involvement is mandatory, even in the absence of signs, systemic symptoms or the presence of negative serology. Ideally, the complementary investigation involves a specialized clinical neurological and cerebrospinal fluid evaluation, if indicated. Complementary tests, such as computed tomography of the skull, chest, and abdomen can be used. The complementary laboratory investigation aims to exclude HIV and HTLV-1 infections and to perform other hematological and biochemical tests, including those for therapeutic monitoring purposes.

### Treatment

Since PCC is an uncommon condition, there are no therapeutic trials gathering evidence to consolidate first- or second-line indications. It is necessary to identify possible aggravating factors in the clinical case being monitored to guide the choice of drug and route of administration, using the following parameters:

I – Those related to the patient in a holistic manner: age, profession, socioeconomic status, nutritional status, and access to medications. Inquire about the presence of comorbidities, current immunosuppressive therapy, and daily medication, and also about the abusive consumption of alcohol or tobacco.

II – Those related to the lesion itself: time of disease, extent of the lesion, presence or absence of necrosis, previous treatments, need or absence for debridement or drainage.

III - Information obtained through imaging tests and by serological, hematological and biochemical laboratory investigations. Based on the above set of data, the definition of mild/moderate or severe condition, according to the perception of the attending physician, will guide the choice of therapy and the need or absence for procedures or even hospitalization. Mild and moderate cases respond satisfactorily to the use of azole derivatives, with fluconazole at a dose of 300 to 400 mg/day for three to six months being effective, as well as itraconazole at a dose of 200 to 400 mg/day, also for three to six months.^40^ Azole derivatives act by inhibiting the CYP-450-dependent enzyme 14-α demethylase and, consequently, by inhibiting the conversion of lanosterol into ergosterol, reducing the formation of the fungus outer cell membrane and its replication.^41^ The clinical response is usually evident at the end of 30 days of treatment and complete healing should occur within six months.^11^, ^12^, ^40^ If the response is slow, it is necessary to check the plasma levels of the azole derivatives in use and verify a possible interference with absorption. It is necessary to bear in mind that azole derivatives are fungistatic drugs and that fungal growth inhibition values ​​obtained in *in vitro* experiments are not necessarily transferable to human patients. Similarly, attention is due to possible drug interactions, since the list of drugs that interact with azole derivatives is not small.^41^ Itraconazole is a potent inhibitor of the CYP3A4 hepatic metabolic pathway, and fluconazole is a potent inhibitor of the CYP2C9 pathway and a moderate inhibitor of the CYP3A4 pathway.^41^, ^42^ Therefore, the predictable drug interaction in solid organ transplant recipients using tacrolimus, sirolimus, or cyclosporine should be considered, as well as patients using warfarin and also taking into account the polypharmacy that elderly patients often use.^41^, ^42^, ^43^, ^44^, ^45^ Itraconazole, in particular, has reduced plasma levels when associated with rifampicin and such interaction can be a cause of therapeutic failure.^40^, ^41^ Itraconazole in particular has increased absorption under acidic pH; therefore, it is best prescribed after large meals accompanied by ingestion of citrus fruits juice.^43^

Severe cases and, exceptionally, moderate cases, require intravenous treatment with fluconazole or amphotericin B deoxycholate or amphotericin B lipid formulation. Amphotericin B is an antibiotic with antifungal properties, derived from *Streptomyces nodosus*, which acts on the ergosterol of the cytoplasmic membrane of the fungal cell with dose-dependent fungistatic or fungicidal properties.^41^, ^46^ It does not promote inhibition of cytochrome P-450; therefore, pharmacological interaction is uncommon. The renal excretion of amphotericin B as an active drug is between 2% and 5% and it is a dialyzable drug, although there is controversy.^46^, ^47^ If using amphotericin B deoxycholate (classical Amph. B), the daily dose used is 0.5 to 1.0 mg/kg/day or on alternate days, restricting the daily dose to a maximum of 50 mg/infusion, regardless of the patients weight. To minimize the pyrogenic effect associated with the release of pro-inflammatory cytokines by classical Amph. B, the initial doses should be lower, for example: 20 mg/day for two days, followed by 30 mg/day for two days and 40 mg/day for two days until stabilizing at the expected daily dose per kilogram of weight.^48^ Classical Amph. B has significant adverse effects, especially renal, resulting in vasoconstriction of afferent arterioles and renal ischemia, inducing renal acidosis, hypokalemia, and hypomagnesemia.^49^, ^50^ Nephrotoxicity is aggravated by the concomitant use of drugs with nephrotoxic potential, particularly the use of non-steroidal anti-inflammatory drugs.^49^, ^50^ Therefore, renal function and electrolytes must be periodically monitored, at the frequency determined by laboratory results. It is mandatory that, before starting treatment with classical Amph. B, the following parameters be predefined: blood count, renal function, electrolytes, liver enzymes, lipase, amylase, and ECG, in addition to other laboratory parameters of interest. Patients with cardiac conduction alterations are at risk and require follow-up with cardiological and electrolyte monitoring on an almost daily basis. To reduce predictable adverse effects, the suggested prescription is: a daily dose of classic Amph. B, with peripheral venous access, diluted in 500 mL of glucose solution (in 5% dextrose, since saline solution precipitates the amphotericin salt).^48^ The following should be added, diluted in each infusion: 50 mg of hydrocortisone, used to eliminate or reduce the pyrogenic effects, and 1000 units of heparin, with the purpose of eliminating or reducing the risks of thrombophlebitis induced by classic Amph. B.^48^, ^51^ After running this combined infusion for six hours, a rapid phase of 250‒500 mL of saline solution is prescribed to partially reduce the effects of renal aggression.^48^, ^49^ Potassium and magnesium (K+ and Mg+) replacement should be performed daily, orally or intravenously, as needed. As a therapeutic alternative, despite its high cost, amphotericin B lipid complex (ABLC) is recommended, which is characterized by good tissue distribution, or liposomal amphotericin B (L-AmB), which is better diffused in the CNS.^52^ These are effective formulations and are indicated for reducing adverse effects, particularly lower nephrotoxicity. Lipid formulations are used via intravenous infusion, previously reconstituted in sterile distilled water and subsequently diluted in 5% dextrose solution, daily and in doses of 3 to 5 mg/kg/day.^49^, ^50^, ^52^, ^53^, ^54^ A promising strategy for the different types of amphotericin B is to reduce the time of therapeutic use of amphotericin to 15 days (as if it were a loading dose) and then transition to an azole derivative, daily and at high doses.^52^, ^55^ This strategy allows the use of higher doses of amphotericin B (classic or lipid complex), for a shorter period of time, with lesser adverse effects and reduces the length of hospital stay. This therapeutic suggestion has already been used in the treatment of paracoccidioidomycosis and histoplasmosis.^52^, ^55^ The combination of amphotericin B deoxycholate and fluconazole to maximize treatment is possible and supported by the literature.^56^ Associated adjuvant interventions, such as debridement of the lesion with removal of necrotic tissue through curettage of the lesion bed, or draining of possible purulent content, have been suggested, but at first, this is an exceptional and restricted conduct.^57^ Similarly, the association of antibiotic coverage is done on a case-by-case basis. If the patient has HIV infection and/or AIDS, it is necessary to consider whether or not to continue with azole derivatives after healing and apparent cure. Regarding histoplasmosis, in patients with AIDS, maintenance with azoles, after apparent cure, can be discontinued if the patient has a CD4 count ≥200 cells/mm^3^.^58^ However, in relation to primary cutaneous cryptococcosis, there is no evidence in this regard in the literature.

### Prognosis

At first, usually evolution under correct treatment is satisfactory and shows resolution, albeit slow in some cases. Response time to treatment will be individual and according to the severity parameters of the case as a whole and the medication used. Cases treated with amphotericin B deoxycholate or lipid formulation should show faster improvement. The same is true with the use of high-dose azole derivatives. It should be considered that depending on the drug and the doses used, adverse effects and/or drug interactions may manifest earlier or later.

In the literature, there are questions about the possible risk of hematogenous dissemination of *Cryptococcus spp*. from the cutaneous PCC lesion or after treatment.^59^ This is a valid question, since it is necessary to take into account the risks associated with systemic corticosteroid therapy, if employed, which is certainly the pharmacological iatrogenesis with the greatest risk for opportunistic infections and to which special attention must be paid.^60^ There are numerous reports that provide reassurance regarding the absence of risks of dissemination of the cutaneous disease, even in immunocompromised patients, as long as they are adequately treated.^11^, ^12^, ^23^, ^24^^,^^27^, ^61^, ^62^, ^63^

However, there is a recent publication in the literature describing the case of a 64-year-old patient who had disseminated and fatal cryptococcosis two years after treatment for PCC diagnosed as a single lesion after trauma in the parietal region. At the time of the cutaneous lesion, no other comorbidities were identified and the patient was considered presumably immunocompetent. And, in the discussion of the case, the authors reported textually: “This is the first case reported of a fatal cryptococcal meningoencephalitis case developing two years after a cured primary cutaneous cryptococcal infection”.^64^ However, in this case report two pieces of information differ from what is expected in cases of PCC: first, the skin lesion was resistant to treatment with up to 600 mg/day of fluconazole, with a MIC of 32 µm/mL, which is unusual in the authors practice and in the literature reports. The patient only responded to liposomal amphotericin B 3 mg/kg/day for one month. Second, the investigation of systemic disease, at the time of the cutaneous event, was limited to chest and abdominal CT, with no mention of neurological, tomographic, cerebrospinal fluid, or serological investigation. These data call into question the mentionthat there was no systemic disease, which had not yet been identified at the time of the initial cutaneous event. Therefore, the authors claim that the case represented systemic disease due to the reactivation of a latent primary cutaneous focus is not entirely unequivocal. An even more challenging case is the report by Amaral et al.^65^ of a 68-year-old patient, a former alcoholic with a smoking history of 100 packs/year, who complained of headache for three days. The patient also had had a lesion on the forearm for a month, which was completely superimposable on semiology to the lesions observed in PCC. During clinical investigation, the cerebrospinal fluid (CSF) showed evidence of lymphocytic meningitis attributed to cryptococcosis and specific serology, by latex agglutination, in the same CSF sample with a titer of 1/1024, findings fully compatible with the diagnosis of meningoencephalitis due to *Cryptococcus spp*. Therefore, depicting a PCC-like cutaneous picture, confirmed by histopathology and culture (*Cryptococcus neoformans*) and with concomitant specific neurological disease.^65^ The only disagreement regarding the interpretation of the case was mentioning that the patient was immunocompetent, when he had been using inhaled corticosteroids for ten years due to chronic obstructive pulmonary disease resulting from smoking. These case reports certainly require paying close attention to details, formulating prognosis on a case-by-case basis, and evaluating the several variables indicated above. In any case, these reports must be mentioned and can be used as a warning regarding prognosis and constitute a significant contribution to the fundamental issue, which is adequate systemic investigation when there is a presumptive diagnosis of primary cutaneous cryptococcosis.

## Conclusion

Primary cutaneous cryptococcosis is an uncommon emerging clinical form, with reports in numerous tropical, subtropical and temperate countries, presented at scientific meetings and dermatology congresses and published in different journals. Clinical suspicion is based on the knowledge that it occurs particularly in immunocompromised individuals but is not exclusive to them. Semiologically, it is polymorphic, with a gelatinous consistency upon palpation, and located in a single exposed area of ​​the body. There are well-established classification and diagnostic criteria, among which culture is the reference method. These criteria must be accurately observed and have to be demonstrated when reporting cases. Treatment is successful with the use of azole derivatives or amphotericin B deoxycholate or amphotericin B lipid formulation. Long-term follow-up is beneficial and recommended.

## Financial support

None declared.

## Authors’ contributions

Silvio Alencar Marques: Drafting and editing of the manuscript; approval of the final version of the manuscript.

Rosangela Maria Pires de Camargo: Approval of the final version of the manuscript.

## Conflicts of interest

None declared.
